# Kinetics of Precipitation Hardening Phases in Recycled 2017A Aluminum Alloy

**DOI:** 10.3390/ma18061235

**Published:** 2025-03-11

**Authors:** Grażyna Mrówka-Nowotnik, Grzegorz Boczkal, Damian Nabel

**Affiliations:** 1 Department of Material Science, Rzeszow University of Technology, Al. Powstancow Warszawy 12, 35-959 Rzeszow, Poland; damiannabel@prz.edu.pl; 2 Faculty of Non-Ferrous Metals, AGH University of Science and Technology, Al. Mickiewicza 30, 30-059 Cra-cow, Poland; gboczkal@agh.edu.pl

**Keywords:** aluminum alloys, precipitation kinetics, activation energy, DSC, recycling

## Abstract

This study investigated the effect of the recycling process on the microstructure, hardness, and precipitation kinetics of strengthening phases in the 2017A aluminum alloy. Light microscopy (LM) and X-ray diffraction (XRD) analyses revealed that the as-cast microstructure of the recycled 2017A alloy contained intermetallic phases, including θ-Al_2_Cu, β-Mg_2_Si, Al_7_Cu_2_Fe, Q-Al_4_Cu_2_Mg_8_Si_7_, and α-Al_15_(FeMn)_3_(SiCu)_2_, and was comparable to that of the primary alloy, confirming its potential for high-performance applications. During solution heat treatment, most of the primary intermetallic precipitates, such as Al_2_Cu, Mg_2_Si, and Q-Al_4_Cu_2_Mg_8_Si_7_, dissolved into the solid Al matrix. DSC analysis of the solution-treated alloy established the precipitation sequence as follows: α-ss → GP/GPB zones → θ″ → θ′/Q′ → θ-Al_2_Cu/Q-Al_4_Cu_2_Mg_8_Si_7_. The combined results from XRD, LM, TEM, and DSC confirmed that both θ and Q phases contributed to strengthening, with θ″ and θ′ phases playing a dominant role. Brinell hardness measurements during natural and artificial aging revealed that hardness increased with aging time, reaching a maximum value of 150.5 HB after ~22 h of artificial aging at 175 °C. The precipitation kinetics of the recycled 2017A alloy was studied via DSC measurements over a temperature range of ~25 to 550 °C, at heating rates of 5, 10, 15, 20, and 25 °C/min. The peak temperatures of clusters, GP zones, and hardening phases (θ′, θ″, θ, and Q) were analyzed to calculate the activation energy using mathematical models (Kissinger, Ozawa, and Boswell). The obtained values of activation energies of discontinuous precipitation were comparable across methods, with values for the θ″ phase of 89.94 kJ·mol^−1^ (Kissinger), 98.7 kJ·mol^−1^ (Ozawa), and 94.33 kJ·mol^−1^ (Boswell), while for the θ′ phase, they were 72.5 kJ·mol^−1^ (Kissinger), 81.9 kJ·mol^−1^ (Ozawa), and 77.2 kJ·mol^−1^ (Boswell). These findings highlighted the feasibility of using recycled 2017A aluminum alloy for structural applications requiring high strength and durability.

## 1. Introduction

Aluminum alloys are extensively utilized as the primary material for highly loaded components in aircraft structures, automotive vehicles, rolling stock, and construction. Their exceptional stiffness-to-weight and strength-to-weight ratios make them ideal for applications where weight reduction is critical. Additionally, their high damage tolerance and inherent resistance to corrosion provide significant advantages over competing materials, enhancing both durability and long-term performance. Furthermore, their excellent manufacturability, including ease of machining, forming, and joining, contributes to their widespread adoption across various industries [1,2,3,4,5,6]. Among aluminum alloys, 2xxx group alloys occupy a special place thanks to their excellent mechanical and technological properties, lightness, and high relative strength (R_m_/δ). Besides Cu as their main alloying element (3.5 to 6% wt.), they also contain Mg, Si, and Mn (up to 1,2%), as well as small amounts of Fe, Ni, Ti, Zr, and Li [1,3,5,7,8,9,10,11,12,13,14,15,16,17,18,19,20,21,22]. They are characterized by high yield strength, good fracture toughness, and excellent fatigue properties, and they also have the properties of heat-resistant materials, owing to the formation of phases rich in Fe, Mn, and Ti [1,2,3,4,5,6,23]. It is not only the good mechanical properties of 2xxx series aluminum alloys that make them attractive, but also the fact that aluminum can be recycled indefinitely without any loss of its properties. Recycling aluminum alloys saves approximately 95% of the energy required to produce the same amount from raw materials. In addition to the environmental benefits, recycling 2017A aluminum alloy, and other aluminum alloys, is economically beneficial. The cost of recycling is much less than producing aluminum from raw materials, leading to cost savings for manufacturers and consumers. Nearly 75% of all aluminum ever produced remains in use today, as it can be endlessly recycled without losing its outstanding properties or quality [1,4,5,7,23,24,25,26,27,28,29,30,31].

The 2xxx series aluminum alloys can be heat-treated to achieve desired properties. However, during recycling, this heat treatment history is lost. The recycled material will need to be re-heat-treated to obtain the expected values. The mechanism responsible for these excellent material properties is the precipitation hardening process [5,7,8,9,10,11,12,13,14,15,16,17,18,19,20]. During aging, secondary intermetallic phases form in the supersaturated alloy and are responsible for precipitation strengthening and play a key role in improving the mechanical properties of 2xxx series aluminum alloys. Many investigations have been devoted to examining the details of the precipitation sequence in Al-Cu alloy [13,14,15,16,17,18,19,20], using both theoretical models and several newly developed techniques [10,14,15,16,17,20].

The findings of numerous studies indicate that the type and volume ratio of the strengthening phases in 2xxx series aluminum alloys are determined primarily by the chemical composition of the alloy and the concentration of the key elements forming these phases (Cu, Mg, and Si). Various research techniques, including TEM, SEM, XRD, and DSC, have been used to investigate and characterize the precipitation sequence and phase composition of these alloys, and the results are widely documented in the scientific literature [17,20]. A review of the literature suggests that depending on the Cu, Mg, and Si contents, as well as the Cu/Mg and Mg/Si ratios, aluminum alloys of the 2xxx series can be strengthened by five key phases: θ (Al_2_Cu), β (Mg_2_Si), S (Al_2_CuMg), Q (Cu_2_Mg_8_Si_6_Al_4_, Al_5_Cu_2_Mg_9_Si_7_, or Al_4_Cu_2_Mg_8_Si_7_), and Si precipitates [14,15,16,17,18]. When the Cu/Mg ratio varies between four and eight, the strengthening occurs mainly by precipitation of the θ (Al_2_Cu) and S (Al_2_CuMg) phases from the solution-treated alloy. The presence of Si significantly affects the precipitation sequence and phase formation. In 2xxx series alloys containing Si, the precipitation of β (Mg_2_Si) and Q (Cu_2_Mg_8_Si_6_Al_4_) phases is promoted alongside the θ (Al_2_Cu) phase. When the Cu content is high and the Mg/Si ratio is larger than one, the β phase can also form in addition to θ. When the Mg/Si ratio is less than one, a Q or S phase can develop, depending on the Si concentration. A very low Si content favors the formation of the S phase, while a higher Si content favors the formation of the Q phase [15,16,22,31].

Application of differential scanning calorimetry (DSC) and the determination of the activation energy of strengthening phase precipitation in aluminum alloys obtained from recycled scrap are of key importance for several reasons. The recycling process can introduce variations in the chemical composition of alloys due to factors such as contamination or oxidation. DSC analysis allows for the assessment of how these factors influence the kinetics of precipitation and the formation of both metastable and stable strengthening phases from the supersaturated alloy. During recycling, impurity elements such as Fe and Si may also accumulate in the alloy, affecting the precipitation of strengthening phases and potentially leading to the formation of brittle, undesirable intermetallic phases. DSC provides valuable insights into how these elements influence the precipitation sequence, enabling the adjustment of processing conditions to minimize their adverse effect on the mechanical properties of the alloys.

Aluminum alloys obtained from recycled scrap often require adjustments in heat treatment parameters, such as solution treatment and aging, to achieve mechanical properties comparable to those of primary alloys. Determining the activation energy of the strengthening phase precipitation process allows for the optimization of time–temperature conditions, ensuring the desired strength parameters.

There is a lack of data in the literature regarding the effect of the recycling process on the precipitation kinetics and activation energy of strengthening phases in the 2017A alloy. Therefore, addressing this topic is well justified. The research conducted in this study fills this gap and demonstrates that the microstructure and properties of the recycled alloy do not differ significantly from those of the primary alloy. Moreover, the determination of activation energy for the precipitation of strengthening phases in the 2017A alloy derived from recycled scrap has not been previously reported in the literature. This study provides new insights into the thermodynamic and kinetic aspects of phase transformations in the recycled alloy, contributing valuable data for optimizing processing conditions and material performance.

Therefore, the aim of this study was to demonstrate the potential of the 2017A aluminum alloy obtained from scrap using the continuous casting process. The study focused on identifying the phase components of the alloy after casting, solution treatment, and aging, based on LM observations and XRD diffractometry. To examine the effect of heat treatment conditions on the mechanical properties of the alloy, precipitation hardening was performed, including solution treatment and both natural and artificial aging at 120 °C and 175 °C. The mechanical properties of the recycled 2017A alloy were evaluated through hardness measurements. Based on DSC calorimetric and XRD diffractometric analyses, the precipitation sequence of strengthening phases in the heat-treated alloy was determined. DSC methods were applied to study the kinetics of precipitation and dissolution of metastable and stable phases in the recycled 2017A aluminum alloy.

Additionally, the activation energies associated with the precipitation of GP zones and the metastable θ″ and θ′ and stable θ (Al_2_Cu) and Q (Cu_2_Mg_8_Si_6_Al_4_) precipitates were calculated using three mathematical models (Kissinger, Ozawa, and Boswell) based on the peak temperatures of exothermic reactions.

## 2. Materials and Methods

The testing material was the aluminum alloy 2017A obtained from recycled materials. The ingot manufacturing process was conducted with a multi-strand continuous casting system, equipped with four oil-lubricated crystallizers. The raw material included big pieces of scrap metal and manufacturing waste in the form of alloy chips 2017A. Approximately 265 kg of scrap was melted in a crucible resistance furnace. Continuous casting of the 2017A alloy was conducted under specific processing conditions. The metal temperature in the furnace was maintained at 730 °C, while in the crystallizer, it ranged from 680 to 700 °C. Cooling was provided by a water flow rate of 25 L per minute, with a casting speed set at 3.5 to 4.0 mm per second. The total cooling water consumption for the four crystallizers was 120 L per minute [23].

Just before casting the ingots, a sample was drawn directly out of the molten bath for examination of chemical composition, which was performed using a The Thermo Scientific ARL-XTRa 3460 spectrometer, Lausanne, Switzerland. Based on the analysis results, necessary adjustments were made to the alloy composition by adding the appropriate amount of the missing elements to ensure compliance with the PN-EN 573-1 standard [32].

### 2.1. Heat Treatment

Heat treatment of the 2017A alloy under study included the precipitation strengthening process. Samples were subjected to heating in an electric resistance furnace until reaching the temperature of 510 °C, at which a homogenous solid α-Al solution exists. They were then annealed for 6 h, and for supersaturation, the alloy was rapidly cooled in water to ~15–20 °C. Natural aging of the alloy was conducted in air at approximately 25 °C for 155 h to achieve the T4 state. In contrast, artificial aging was performed at 120 °C and 175 °C for the same duration, leading to the T6 state. Following artificial aging, the samples were quenched in water.

### 2.2. Hardness Test

The hardness of the 2017A alloy was measured immediately after supersaturation and during natural aging at 25 °C, as well as during artificial aging at different temperatures (120 °C and 175 °C). Hardness measurements were carried out using an Instron Wolpert hardness tester (High Wycombe, UK) with the Brinell method, using a 62.5 kg load and a 2.5 mm diameter indentation ball. Hardness measurements were performed on at least three different samples in each condition, and the reported values are average values. HBW 2.5/62.5 hardness measurements of supersaturated and aged samples at different temperatures and times were performed continuously. From the results, the average hardness was calculated and plotted as a function of aging time.

### 2.3. Microstructural Examination

Microscopic analysis of the alloy following casting, solution treatment, and aging was carried out using a Leica DMI 3000M light microscope, Wetzlar and Mannheim, Germany and a Jeol-2100 transmission electron microscope, Musashimurayama, Japan. The samples were cut with a Discotom-6 precision cutting machine and mounted in Bakelite. They were then ground with SiC papers of 250, 500, 800, 1000, and 1200 grit and polished with 3 µm and 1 µm diamond polycrystalline suspensions. The final polishing step was carried out using Al_2_O_3_ suspension. Microstructure observation was carried out on polished samples, which were then etched in Keller’s reagent: 2 cm^3^ of HF + 3 cm^3^ of HCl + 20 cm^3^ of HNO_3_ + 175 cm^3^ of H_2_O. Electrochemical polishing was used to prepare the thin foils in a reagent mixture of 260 mL of CH_3_OH + 35 mL of glycerol + 5 mL of HClO_4_ using a Tenupol-3 polisher and Cresington 108 automatic sputter coater.

The phase components of the 2017A alloy microstructure after casting, solution heat treatment, and natural aging were qualitatively analyzed on solid samples with a ground and polished surface. To ensure that the observed alloy phase components were reproducible for an alloy in the same state, XRD analyses for each alloy state were carried out on at least two samples. The phase composition was determined using an ARL XTRa X-ray diffractometer from Thermo Fisher, Waltham, MA, USA. A filtered copper lamp (CuKα, λ = 0.1542 nm) was used, with a voltage of 40 kV, a current of 30 mA, a range of 2θ = 20–50°, and a step size of 0.02°/6 s. The phase composition was determined using the Powder Diffraction File (PDF), developed and issued by the ICDD (International Center for Diffraction Data).

### 2.4. Thermal Analysis

To analyze the thermal effects associated with the precipitation and dissolution of strengthening phases in the 2017A alloy, DSC examinations were conducted immediately after quenching the samples from the solutionizing temperature. The DSC measurements for cast and solution heat-treated specimens were performed with the SETARAM SETSYS Evolution-1200 thermal analyzer. The samples were prepared in a disk shape with 3 mm diameter, 1.5 mm thickness, and an approximate mass of 25–28 mg. An argon protective atmosphere was used to prevent oxidation of the samples during multiple DSC analyses. DSC experiments were conducted at least twice for each heating rate. To determine the sequence of precipitation of strengthening phases from the supersaturated alloy, one of the solution-treated specimens was heated at a rate of 10 °C/min from room temperature to 700 °C using a DSC analyzer. However, to determine the activation energy, the remaining samples were heated from 25 °C to 550 °C with different heating rates of 5, 10, 15, 20, and 25 °C/min. The peak temperatures (*T_p_*) of the hardening phases from each heating rate were measured and collected to determine the kinetic parameters of the 2017A aluminum alloy. Three mathematical models (Kissinger, Ozawa, and Boswell) were used to determine the activation energy [33,34,35,36,37,38,39,40,41,42,43,44,45]. Investigation of kinetics transformation (precipitation/dissolution of the precipitates) is always related to the concept of activation energy. Studies of precipitation processes are associated with nucleation and growth processes, which dominate in supersaturated alloys. In general, separate activation energies must be identified for individual nucleation and growth steps during a transformation, although they are usually combined into a single activation energy representing the overall precipitation process [40]. In the present study, a non-isothermal method was applied, in which the samples were heated at a constant rate q, and the heat evolved was recorded as a function of temperature or time.

The study of discontinuous precipitation kinetics of the hardening phases under non-isothermal conditions is based on the Johnson–Mehl–Avrami (JMA) [34,35,36,37,38,39,40,41,42,43,44,45] Equation (1) of isothermal transformation kinetics:

(1) $$y\left(t\right)=1-exp(-kt^{n}),$$

where *y*(*t*) is the volume fraction of the initial material transformed at time *t*, *n* is the Avrami exponent (which reflects the nucleation rate and the growth morphology), and *k* is the reaction rate constant, which can usually be derived from an Arrhenius equation in Equation (2):

(2) $$k=k_{0}\exp\left(-\frac{E_{a}}{RT}\right),$$

where $E_{a}$ is the activation energy for the crystallization reaction, which describes the overall precipitation process, R is the universal gas constant (8.314 J mol^−1^ K^−1^), T is the isothermal temperature, and $k_{0}$ is the frequency factor.

The theoretical basis for interpreting the DSC results at different heating rates involved three mathematical models—Kissinger, Ozawa, and Boswell—which were used to analyze and determine the activation energy of the hardening phases in the AA 2017A aluminum alloy, as shown in Equations (3)–(5), respectively:

(3) $$\mathrm{Kissinger}’s\;\mathrm{model}:\;Y=\ln(\frac{q}{T_{p}^{2}})=-\frac{E_{a}}{RT_{p}}+C_{1},$$

(4) $$\mathrm{Ozawa}’s\;\mathrm{model}:\;Y=\ln(q)=-\frac{E_{a}}{RT_{p}}+C_{2},$$

(5) $$\mathrm{Boswell}’s\;\mathrm{model}:\;Y=\ln(\frac{q}{T_{p}})=-\frac{E_{a}}{RT_{p}}+C_{3},$$

where $C_{1}$, $C_{2}$, and $C_{3}$ are constants, $T_{p}$ is the temperature at the maximum peak of the hardening phases, q=dT/dt is the heating rate, and $E_{a}$ is the activation energy. Y is an assessment of thermal analysis for each mathematical model.

## 3. Results

The spectrometric analysis of the chemical composition of the ingots obtained from recycled scrap confirmed that the content of alloying elements met the specifications set for the 2017A alloy. The chemical composition of the investigated alloy is presented in Table 1.

**Figure 1:**
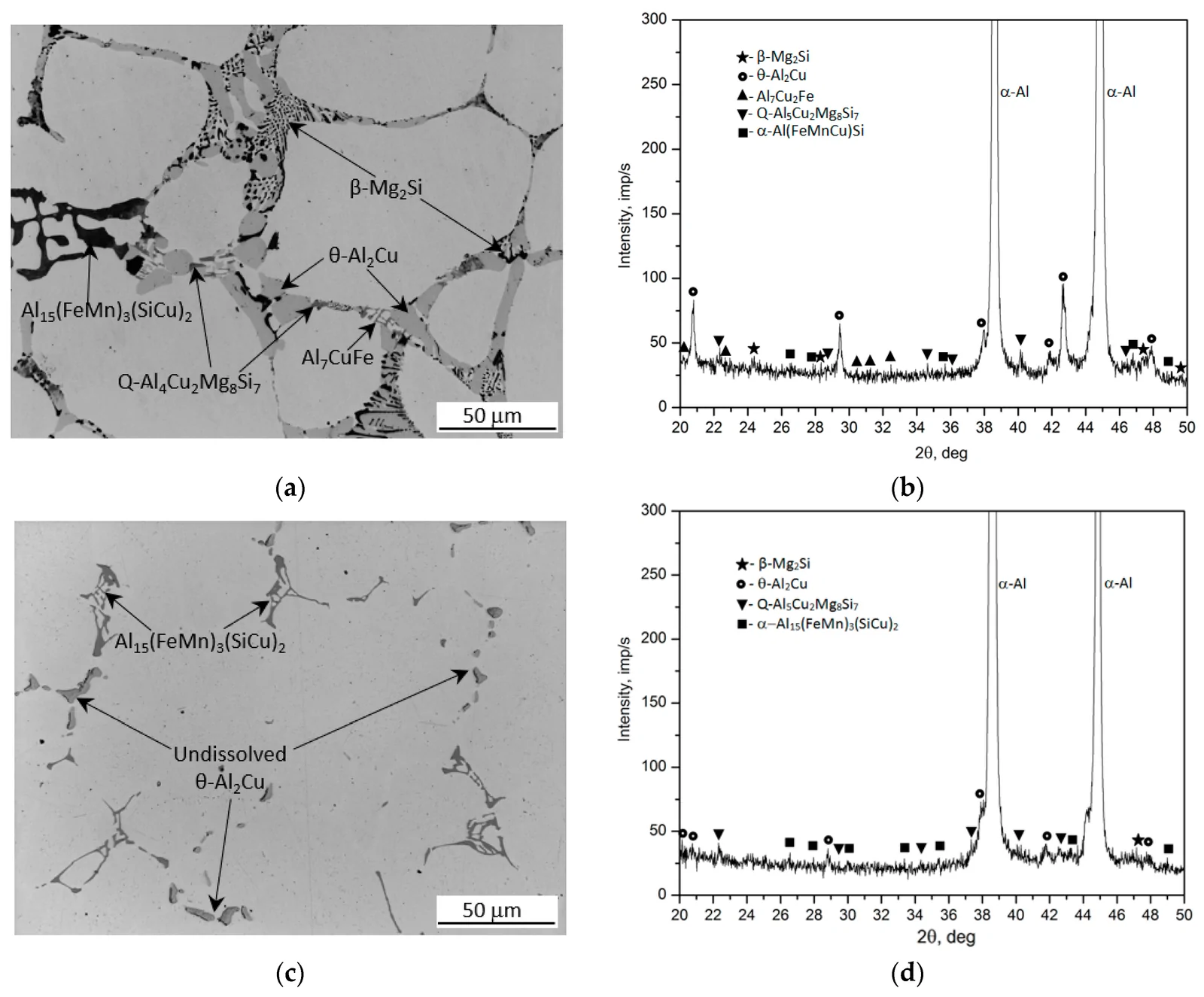

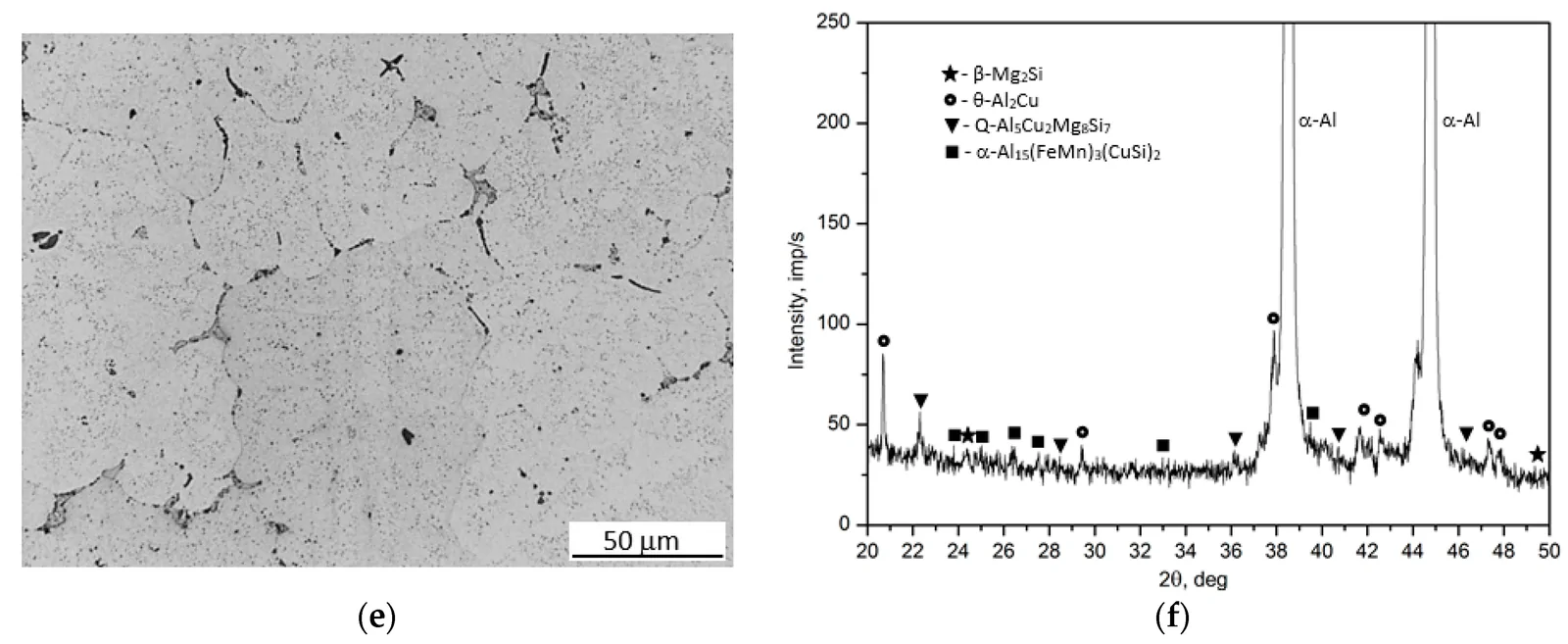
As-cast microstructures and X-ray diffraction pattern of the 2017A alloy: (**a**) LM and (**b**) XRD; after solution heat treatment at 510 °C/6 h: (**c**) LM and (**d**) XRD; and alloy after artificially aging for 55 h at 175 °C: (**e**) LM and (**f**) XRD.

Figure 1 shows the microstructure (LM) and phase composition (XRD) of recycled 2017A aluminum alloy in the as-cast state (Figure 1a,b), supersaturated solid solution state (Figure 1c,d), and supersaturated plus naturally aged state at ~25 °C for 155 h (Figure 1e,f). Based on previous examinations and results [23] from microscopic observations as well as XRD studies, it was demonstrated that the as-cast microstructure of the 2017A alloy obtained from recycling consists of the following phases: binary θ-Al_2_Cu and β-Mg_2_Si phases, a ternary Al_7_Cu_2_Fe phase, quaternary Q-Al_4_Cu_2_Mg_8_Si_7_ phase, and quinary α-Al_15_(FeMn)_3_(SiCu)_2_ phase. Analysis of these results shows that the largest relative volume is occupied by precipitates of the binary θ-Al_2_Cu phase (Figure 1a,b). The XRD spectrum (Figure 1b) exhibited the highest number of high-intensity reflections originating from this phase. Microscopic observations (Figure 1c) and XRD analysis (Figure 1d) of the solution heat-treated samples at 510 °C showed that during annealing for 6 h, most of the primary precipitates of Al_2_Cu, β-Mg_2_Si, and Q-Al_4_Cu_2_Mg_8_Si_7_ intermetallic phases observed after casting (Figure 1) were dissolved in the solid α-Al solution (Figure 1c,d). However, the morphology of the remaining undissolved intermetallic precipitates, mainly those containing Fe, also changed. It was found that during annealing to supersaturation, the edges of the α-Al_15_(FeMn)_3_(SiCu)_2_ phase particles, which appeared in the cast state in the form of Chinese script, became rounded. Additionally, during annealing at 510 °C for six hours, the lamellar and needle-like particles of the Al_7_Cu_2_Fe phase transformed into spheroidal-like particles of the α-Al_15_(FeMn)_3_(SiCu)_2_ phase (Figure 1c). The microstructure of the 2017A alloy after artificially aging for 55 h at 175 °C exhibited very fine, dispersive precipitates homogeneously distributed throughout the alloy volume (Figure 1e). A prolonged aging time leads to the precipitation of stable equilibrium phases, their growth, and coagulation. Based on XRD diffractometric results (Figure 1f), previous studies [23], and the literature data [5,15,33], it can be stated that the main phases of strengthening in the 2017A alloy are the θ-Al_2_Cu and Q-Al_4_Cu_2_Mg_8_Si_7_ precipitates.

**Figure 2:**
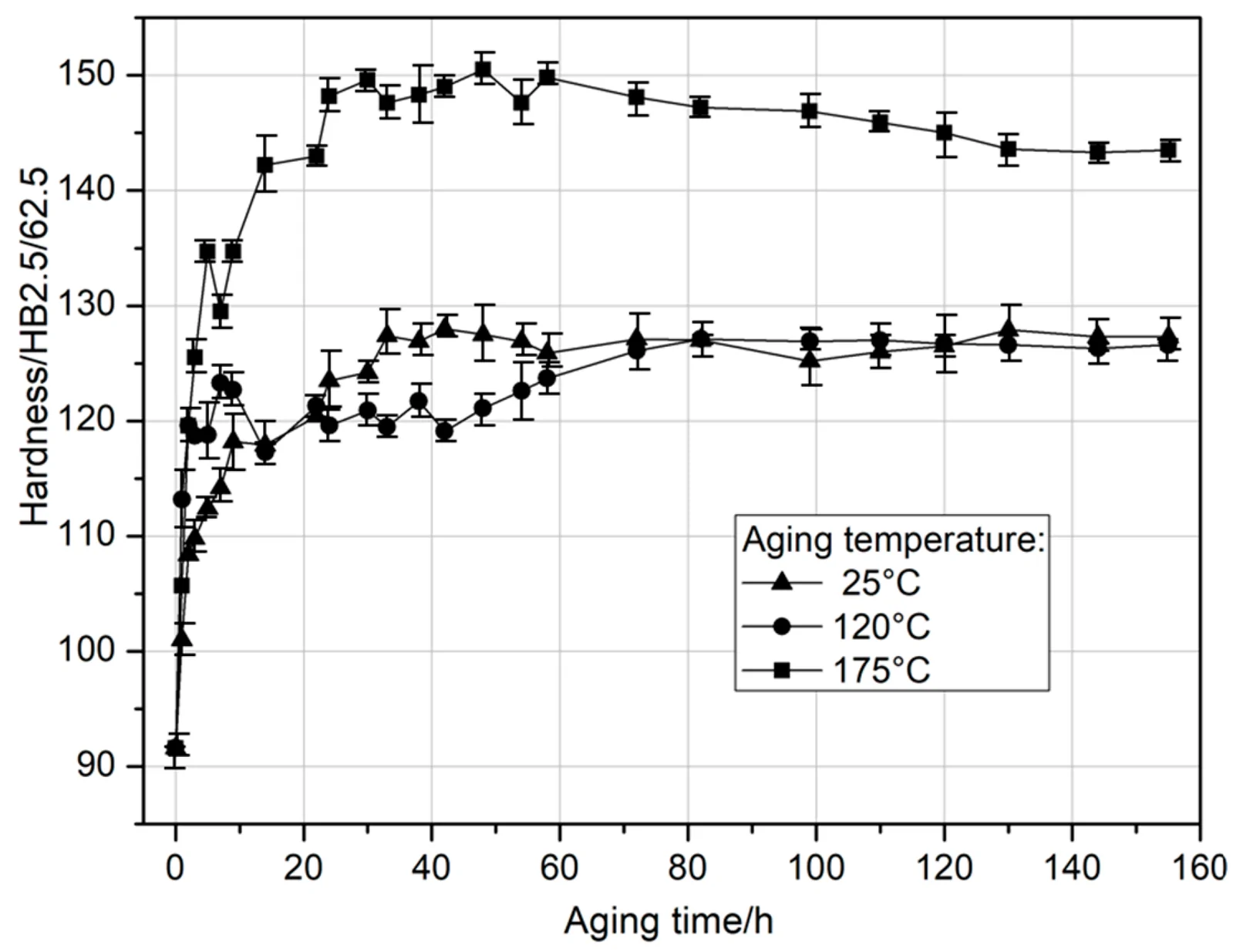
Influence of temperature and aging time on the evolution of hardness of an alloy subjected to solution heat treatment at 510 °C and natural and artificial aging.

Solution heat-treated samples were natural aged at 25 °C and artificially aged for 155 h at 120 °C and 175 °C. During aging, hardness values were monitored and plotted in Figure 2. From the initial hardness of around 91 HB in the solution-treated state, the hardness of the material increased with aging time. The first small hardness peak (118.2 HB) appears on the curve after approximately 8 h of natural aging. Continued aging causes a slight decrease in hardness, followed by a continuous increase with further aging duration until reaching a maximum value of 128.0 HB after 42 h. The tested alloy, having reached its peak hardness during natural aging, does not overage, which is usually characterized by a decrease in hardness. The aging curve shows a plateau—a range of hardness that has stabilized. Hardness values close to the maximum remain on a comparable level throughout 155 h of natural aging. Aging at 120 °C results in a level of hardness closely corresponding to that achieved through natural aging. Samples aged at the highest temperature (175 °C) exhibit the highest hardness values (150.5 HB), which are achieved after approximately 22 h of artificial aging (Figure 2).

**Figure 3:**
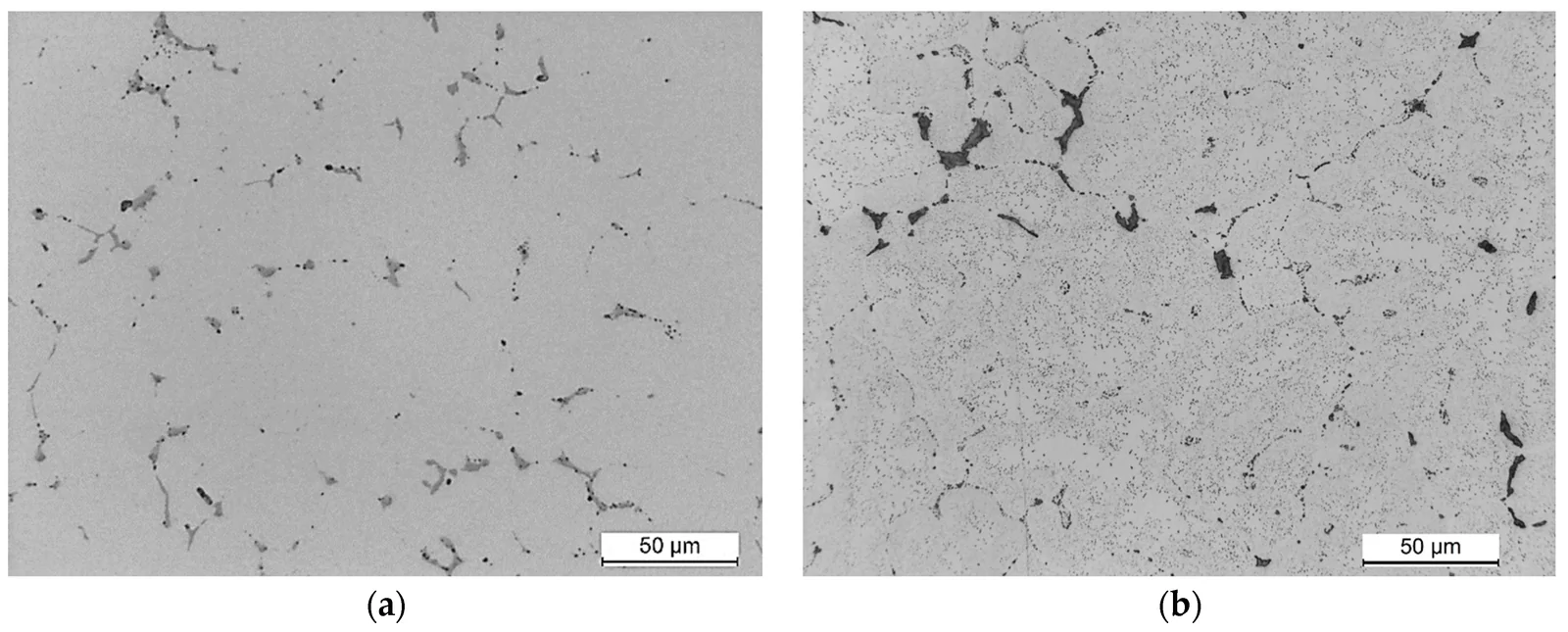
Microstructure of aluminum alloy 2017A after aging: (**a**) at a temperature of 25 °C for 40 h and (**b**) 175 °C for 65 h.

The microstructure of the 2017A alloy, aged naturally to peak hardness, shows only undissolved particles of primary intermetallic phases. However, no secondary strengthening phase particles are observed (Figure 3a). Increasing the temperature to 175 °C and aging time to 65 h resulted in the formation of very fine secondary hardening precipitates—θ-Al_2_Cu and Q-Al_4_Cu_2_Mg_8_Si_7_—which are dispersed and evenly distributed through the alloy volume (Figure 3b).

**Figure 4:**
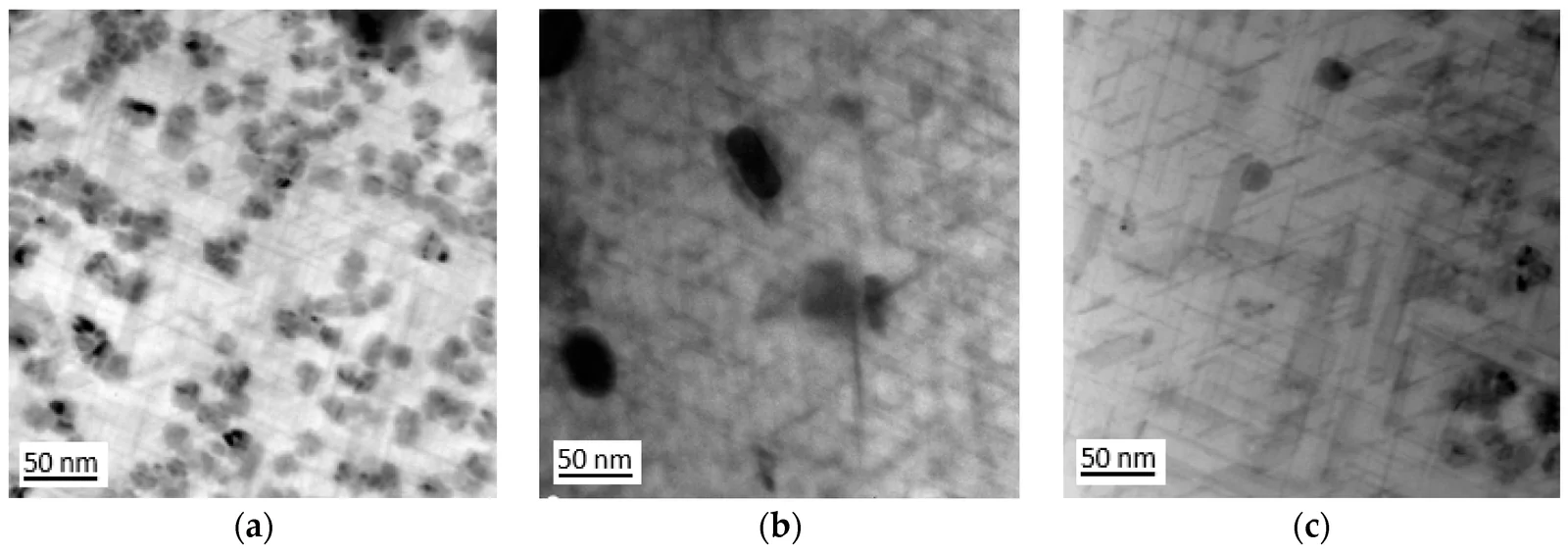
Microstructure of the 2017A alloy after aging at a temperature of 175 °C for (**a**) 5 h and (**b**,**c**) 22 h, showing precipitates of strengthening phases θ′, θ′, and Q′.

To observe the θ-Al_2_Cu and Q-Al_4_Cu_2_Mg_8_Si_7_ phases’ precipitations in the specimen artificial aged at 175 °C for 5 and 22 h with the maximum hardness value, TEM analysis was performed, as shown in Figure 4. After 5 h of aging, very fine needle-like metastable transition phases—θ″ and θ′ as well as Q′—were observed, embedded within the aluminum matrix, with an average length of approximately 50 nm. Extending the aging time to 22 h increased the length of the needle-like precipitates to approximately 100–150 nm (Figure 4b,c).

### DSC Examination

**Figure 5:**
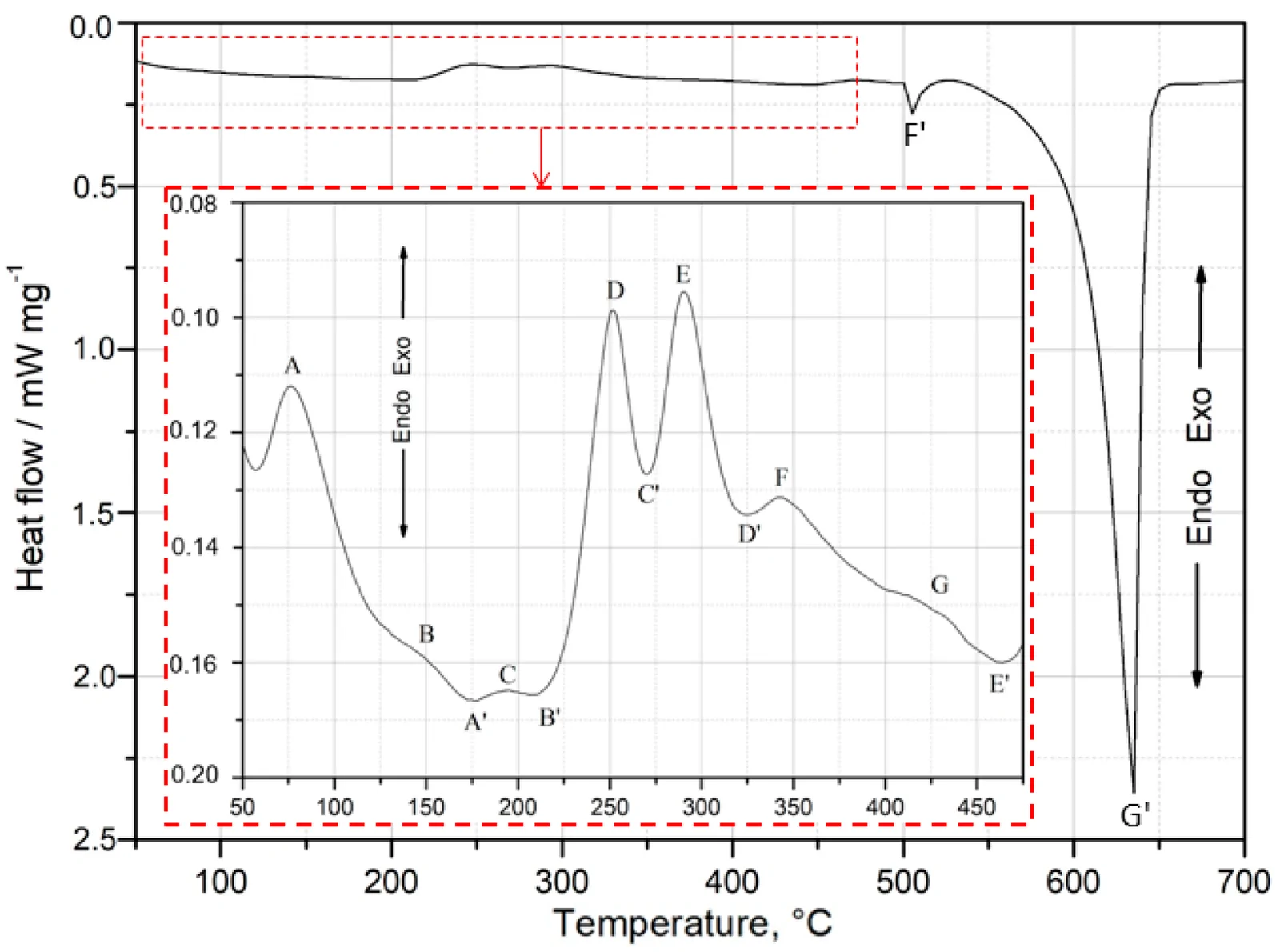
The DSC graph of as-quenched of 2017A alloy specimen solution heat-treated and heated up to 700 °C at a rate of 10 °C min^−1^.

Figure 5 presents the DSC curves obtained during heating at q = 10 °C min^−1^ in the calorimeter for the as-quenched specimen of the 2017A alloy. Six exothermic and six endothermic peaks were identified from the DSC thermogram. The characteristic temperature values of the exo- and endothermic peaks recorded on the DSC curve are presented in Table 2.

Exothermic peaks are indicated with capitals A–G, while endothermic peaks are denoted by capitals with a prime symbol (A′–G′). The part of the DSC curve within the temperature range of 50 °C to 475 °C, where precipitation of the strengthening phases was observed during continuous heating, has been magnified (Figure 5). Based on the DSC, XRD, and hardness test results, an attempt was made to identify the precipitation sequence and provide an explanation for the origin of the main peaks. The results (Figure 1, Figure 2, Figure 3, Figure 4 and Figure 5) indicate that during continuous heating in the calorimeter of the supersaturated 2017A aluminum alloy, strengthening phases—primarily θ-Al_2_Cu, and to a lesser extent Q-Al_4_Cu_2_Mg_8_Si_7_—are precipitated. The precipitation sequence of the hardening θ-Al_2_Cu and Q-Al_4_Cu_2_Mg_8_Si_7_ phases was recorded on the DSC curve (Figure 5).

Based on the DSC curve (Figure 5), it can be presumed that the hardening process begins with the formation of Cu and Mg atom co-clusters, corresponding to exothermic peak A between 61 and 95 °C (Table 2) [8]. As the temperature increases, GP zones (exothermic peaks B and C) form coherently with the matrix, followed by their dissolution (endothermic peaks A′ and B′). Exothermic peaks of the highest intensity—D (between 239 and 265 °C)—are due to precipitation of the θ″ phase, while E (between 273 and 311 °C) corresponds to the formation of the θ′/Q′ phases, which exhibit partial coherence with the alloy matrix. The precipitation of these phases is responsible for achieving the maximum mechanical properties of the alloy [8,13,14,15,16,17,18,23]. Continued heating leads to the dissolution (endothermic peaks C′ and D′) of the metastable θ′ and Q′ phases. Finally, the last exothermic peaks—F and G, in the temperature ranges of 329–367 °C and 401–444 °C, respectively—indicate the formation of stable θ-Al_2_Cu and Q-Al_4_Cu_2_Mg_8_Si_7_ precipitates, which are incoherent with the matrix. Upon further heating of the supersaturated sample, a sharp endothermic peak F′ is observed between 508 and 522 °C on the DSC graph. This effect (F′) corresponds to the dissolution of all intermetallic phases. Continued heating of the alloy ultimately leads to the dissolution of the α-Al phase, as indicated by endothermic peak G′.

**Figure 6:**
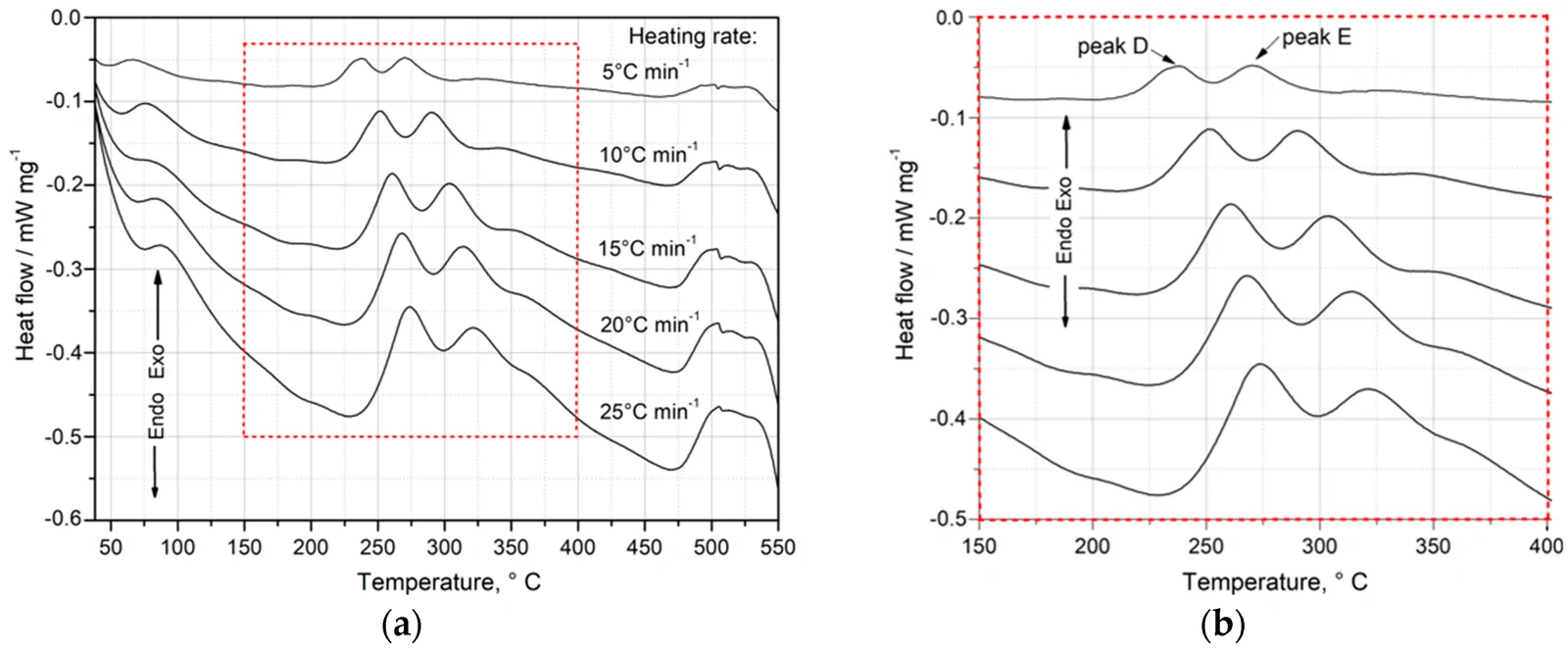
DSC curve showing the change of heat flow vs. temperature during heating of solution-treated samples at different heating rates: 5, 10, 15, 20 and 25 °C·min^−1^ over the temperature range of: (**a**) 25 to 505°C; (**b**) enlarged section of the DSC in the temperature range of 150 to 400°.

In order to define the activation energy (*E_a_*) of the precipitation-hardening phases in the supersaturated recycled 2017A alloy, the DSC results were analyzed. Figure 6 presents the DSC curves obtained at various heating rates (*q)* from 5 to 25 °C/min^−1^, starting from room temperature up to 550 °C. The thermal effects related to the precipitation and dissolution transformations were recorded (Figure 6).

**Figure 7:**
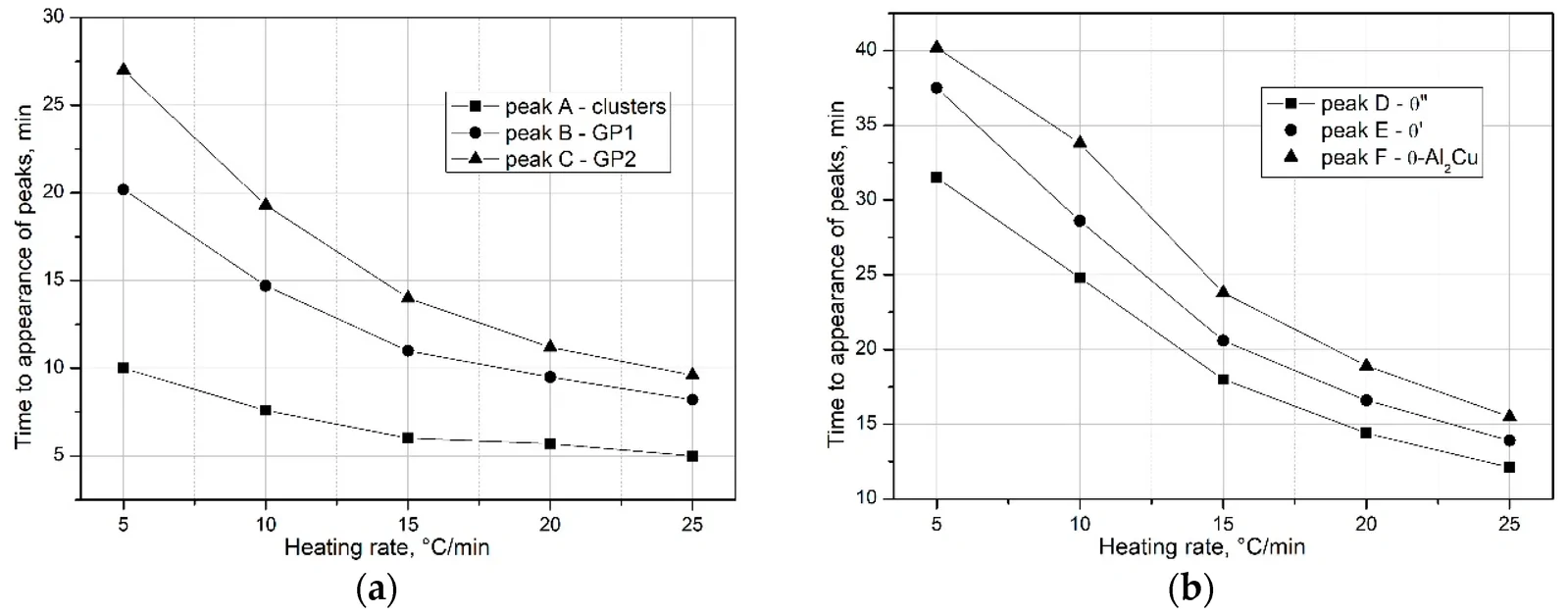
Influence of heating rate versus time to appearance of exothermic peaks associated with precipitation of metastable: (**a**) clusters, GP1, and GP2 zones, and (**b**) transition phases θ″, θ′, and Q′ and stable phase θ-Al_2_Cu.

Analysis of the obtained DSC findings shows that an increase in the heating rate (*q*) affects the kinetics of the precipitation of strengthening phases in the supersaturated 2017A alloy. As shown in Figure 6 and Figure 7, an increase in the heating rate (q) reduces the time required for the occurrence of peaks—precipitation of clusters, GP zones (Figure 7a), and metastable transition phases (θ″ and θ′), as well as the stable θ-Al_2_Cu phase on the DSC curves (Figure 7b).

The heating rate (*q*) also influences the peak temperatures $T_{p}$ relating to precipitation of strengthening phases in the supersaturated 2017A alloy (Table 3).

**Figure 8:**
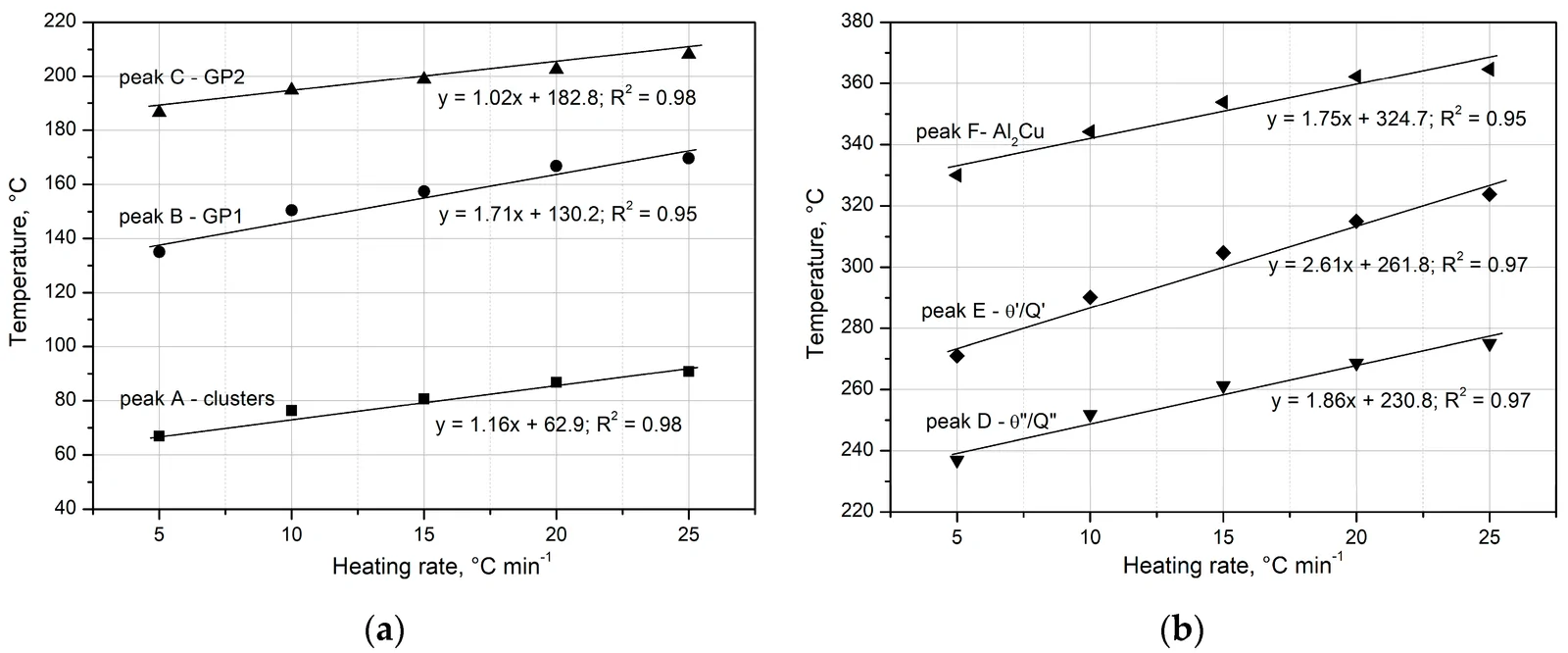
Influence of heating rate on the temperature of precipitation of (**a**) clusters, GP1, and GP2, and (**b**) metastable transition phases θ″, θ′, and Q′ and stable θ-Al_2_Cu phase.

The T_p_ values of the exothermic and endothermic peaks in the DSC thermograms shift to higher temperatures as the heating rate q rises (Figure 6, Figure 7 and Figure 8, Table 3). The high correlation coefficients (R^2^ = 0.95 ÷ 0.98) indicate a strong dependence of the temperature at which strengthening phases are released on the heating rate (Figure 8a,b). The observed dependence of exothermic peak temperatures in reactions A–F on the scanning rates indicates that these processes are thermally activated. The influence of the heating rate may be attributed to a reduction in the number of Cu atoms precipitated, resulting from their increased solubility in the solid state at higher precipitation temperatures when a higher heating rate is used [21,22,23]. This effect can also be attributed to the diffusive nature of the precipitation reactions.

**Figure 9:**
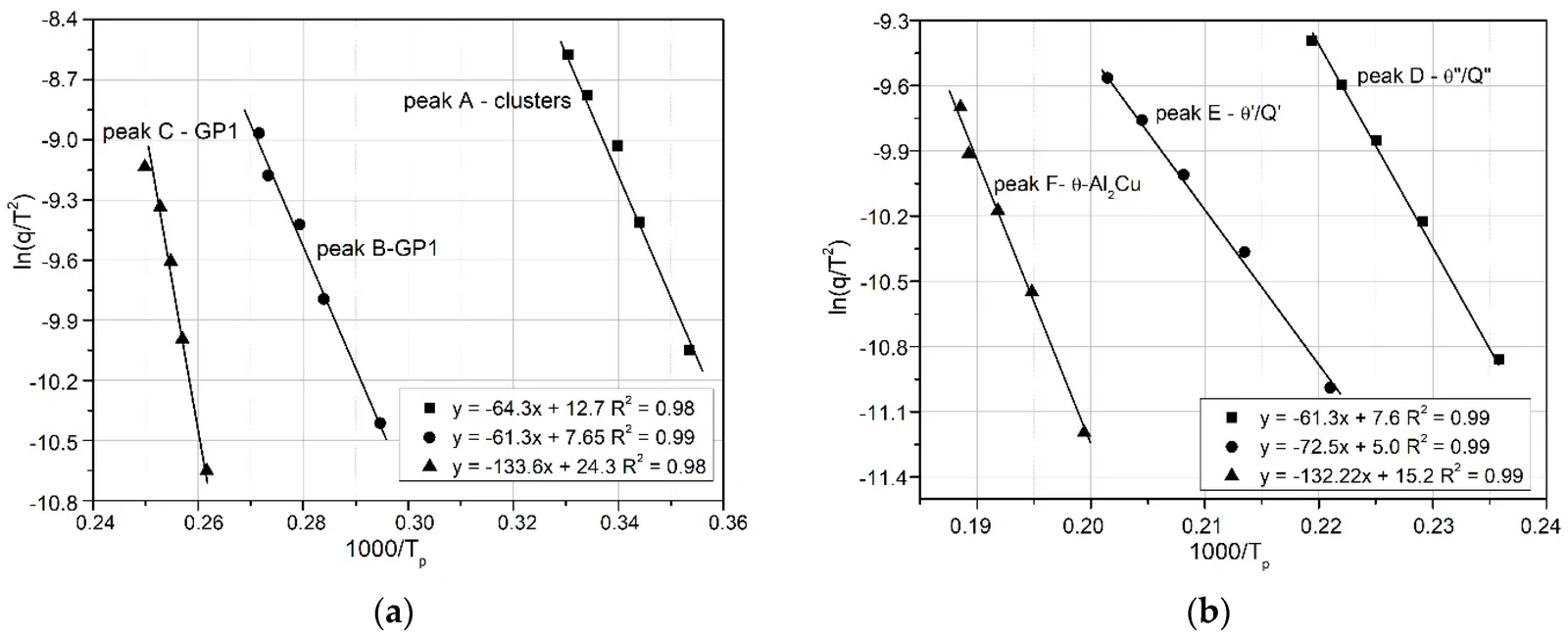
Kissinger’s graphs for the relationship of ln(q) vs. 1000/RT related to the observed heat effects associated with the precipitation processes of: (**a**) clusters, GP1, GP2; and (**b**) θ″/Q″, θ′/Q′ and Q phases in the 2017A aluminum alloy.

**Figure 10:**
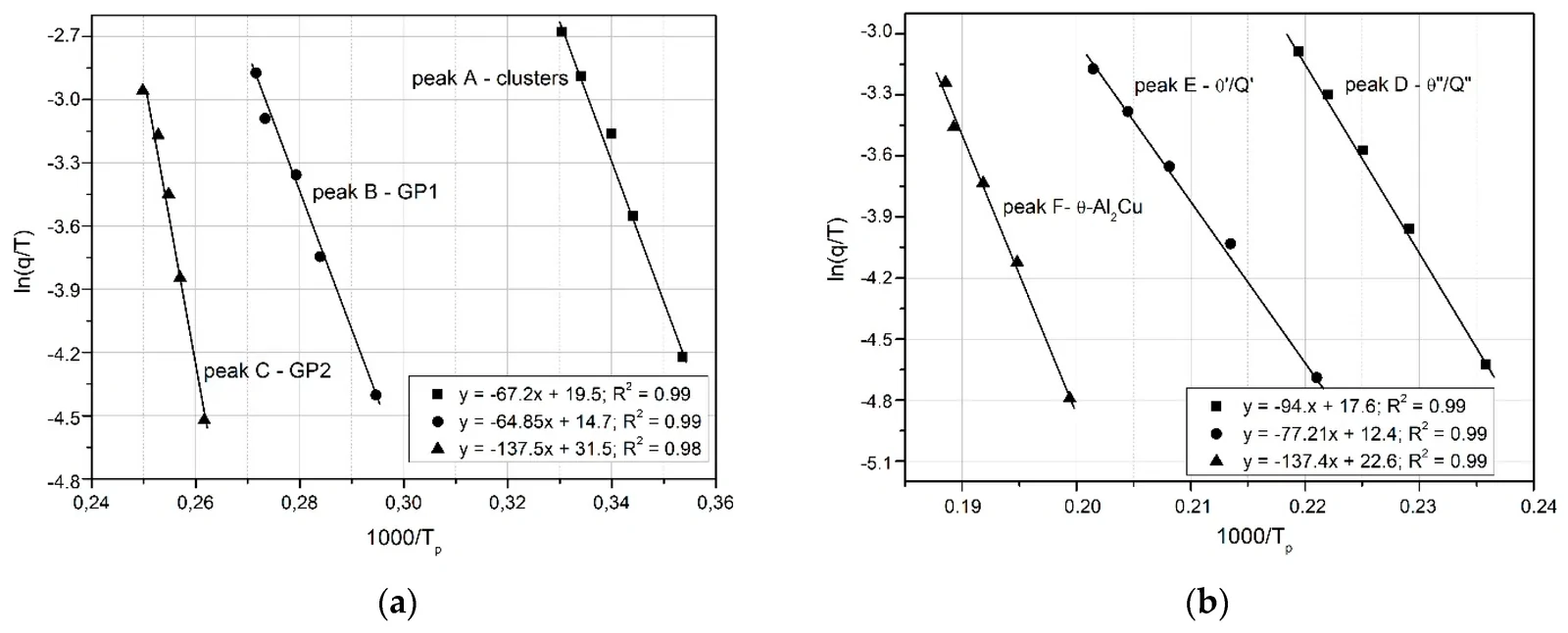
Boswell’s graphs for the relationship of ln(q) vs. 1000/RT related to the observed heat effects associated with the precipitation processes of: (**a**) clusters, GP1, GP2; and (**b**) θ″/Q″, θ′/Q′ and Q phases in the 2017A aluminum alloy.

**Figure 11:**
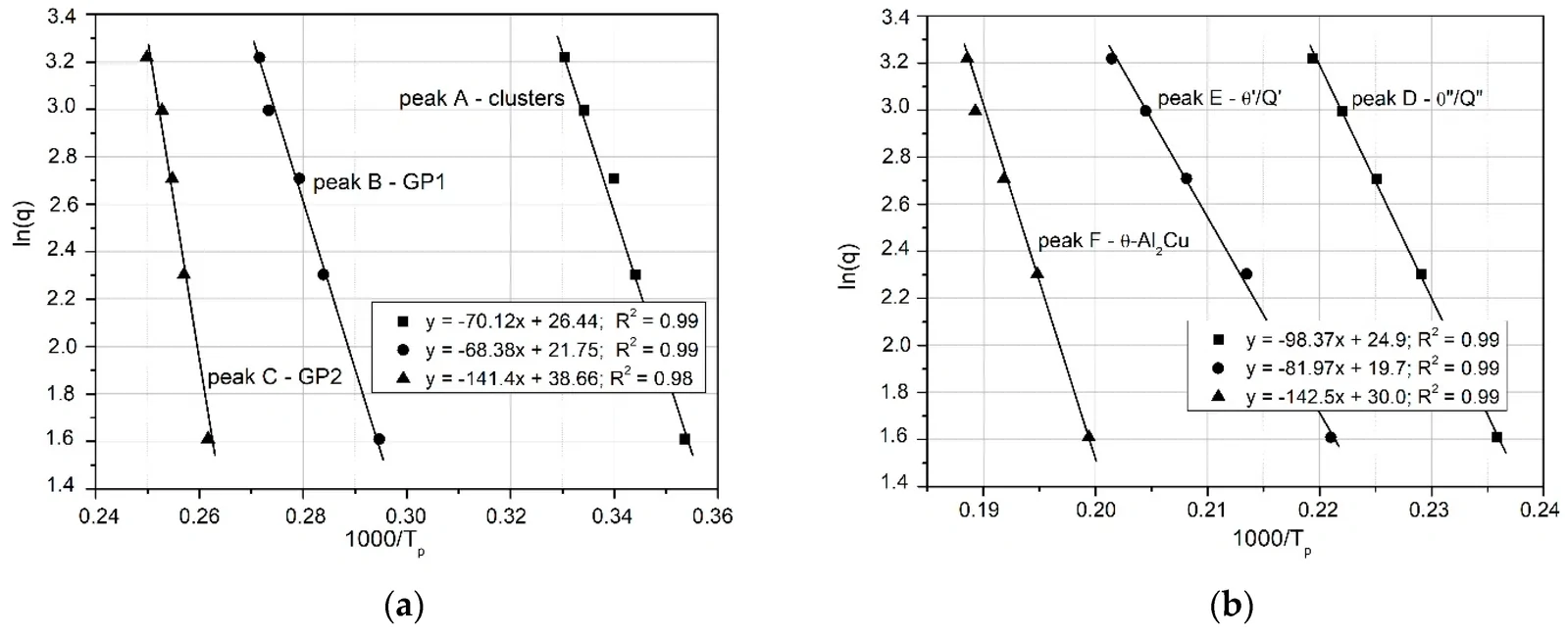
Ozawa’s graphs for the relationship of ln(q) vs. 1000/RT related to the observed heat effects associated with the precipitation processes of: (**a**) clusters, GP1, GP2; and (**b**) θ″/Q″, θ′/Q′ and Q phases in the 2017A aluminum alloy.

The activation energy of the clusters, GP zones, and hardening phases (θ and Q) in the 2017A alloy was determined by analyzing the shift in the temperature of the maximum exothermic peaks $T_{p}$ as a function of the heating rate q. Using Equations (3)–(5), diagrams of Y versus $1000/T_{p}$ were plotted, as illustrated in Figure 9, Figure 10 and Figure 11:

- Kissinger method $\ln(q/T_{p}^{2})$ vs. $1000/T_{p}$(Figure 9),
- Boswell method $\ln(q/T_{p})$ vs. $1000/T_{p}$ (Figure 10),
- Ozawa method ln⁡(q) vs. $1000/T_{p}$ (Figure 11).

**Figure 12:**
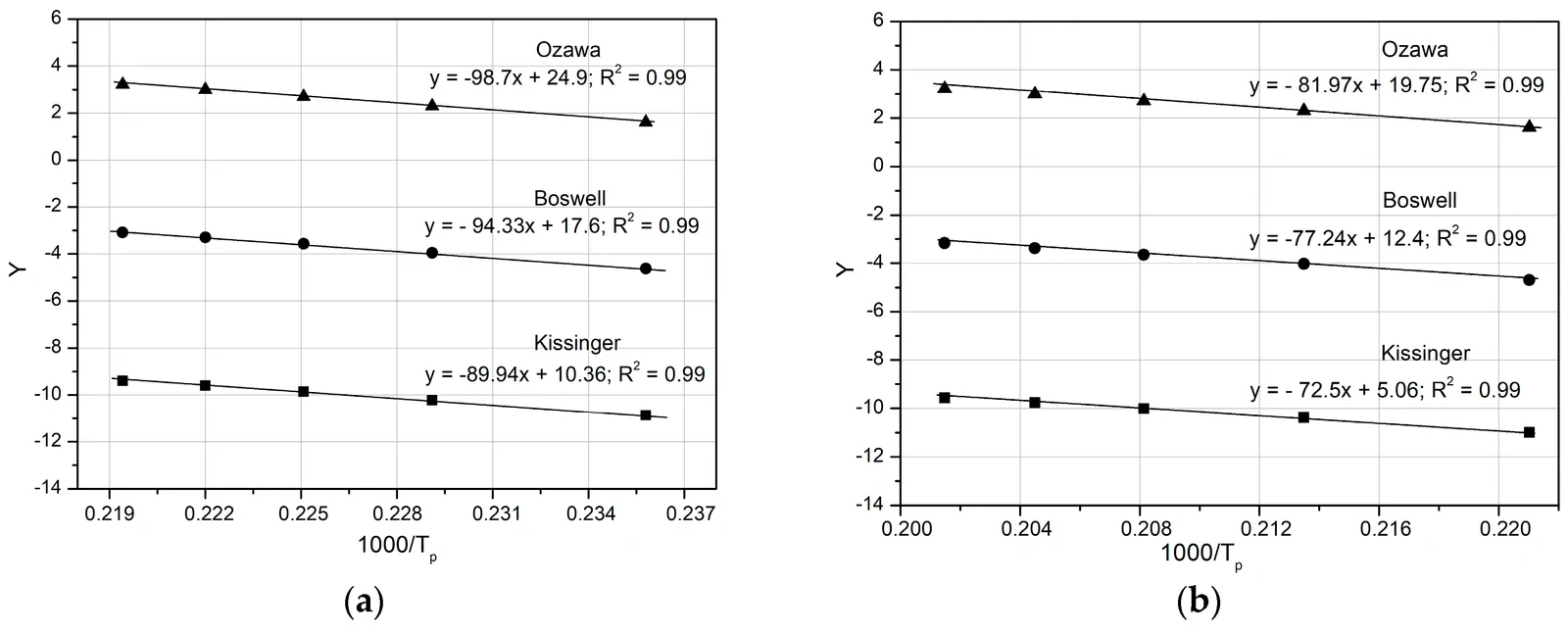
Plot of Y versus 1000 R/T for three mathematical models: (**a**) precipitation of θ″ (peak D) and (**b**) precipitation of θ′/Q′ (peak E).

**Figure 13:**
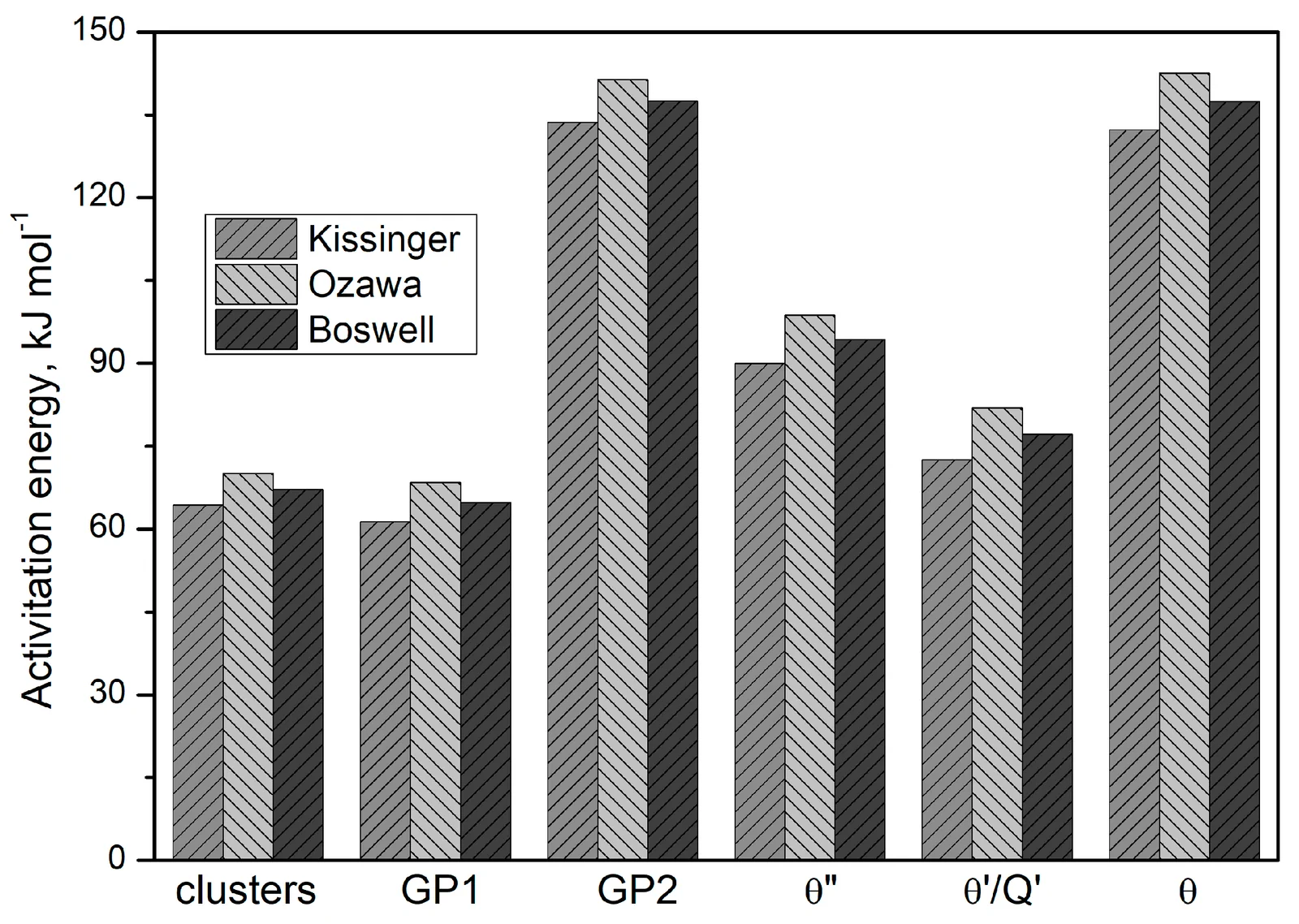
Activation energy determined by the Kissinger, Ozawa, and Boswell approaches.

Figure 12 compares the Y versus 1000 R/T curves for θ″ (peak D) and θ′ (peak E), obtained using the three mathematical models: Kissinger, Ozawa, and Boswell. The activation energy values, derived from the slopes of the curves, are very similar (e.g., for the θ″ phase: Kissinger, 89.94 kJ mol^−1^; Ozawa, 98.7 kJ mol^−1^; Boswell, 94.33 kJ mol^−1^), as shown in Table 4 and Figure 13.

Based on these results (Table 4 and Figure 13), it may be concluded that the activation energies obtained from the three mathematical models differ insignificantly, including for clusters, GP zones, and stable precipitates (θ and Q phases). The Ozawa equation yields the highest activation energy value (Table 4 and Figure 13).

## 4. Summary

The obtained results demonstrated that it is possible to produce high-quality alloy ingots from scrap through continuous casting, confirming the potential for utilizing secondary materials in the production of high-quality alloys. Microstructure analysis revealed that the structure of the recycled alloy does not significantly differ from that of the alloy obtained from primary components and is comparable to the microstructure found in other studies on alloys from the 2xxx series [4,9,14,28,29,46,47,48,49,50,51,52,53,54].

Differential scanning calorimetry (DSC) studies showed that despite the presence of trace amounts of elements such as Fe and Si, the kinetics of precipitation of metastable and stable strengthening phases did not undergo significant changes. It was confirmed that the sequence of precipitation of strengthening phases in the recycled alloy is consistent with that observed in the primary alloy [11,14,15,16,17,18,19,49,53,54]. Properly selected heat treatment parameters allowed for the obtention of a microstructure and mechanical properties comparable to the primary alloy, meeting the requirements for this alloy grade [2,4,7,19,23,25], indicating its effective use in applications requiring high mechanical properties. During the natural and artificial aging of the supersaturated 2017A alloy, an increase in Brinell hardness (HB) was observed with increasing aging time. The highest hardness was achieved for the alloy subjected to artificial aging at 175 °C, reaching a value of 150.5 HB. In the case of aging at 120 °C, the hardness remained at a level similar to that obtained in the natural aging process (approximately 128 HB). The precipitation kinetics characteristics were determined using DSC under non-isothermal conditions. It was found that the heating rate (*q*) shortens the time to thermal peaks and affects the peak temperatures ($T_{p}$) of strengthening phase precipitation in the supersaturated 2017A alloy. The $T_{p}$ values for exothermic and endothermic peaks in the DSC thermograms shift toward higher temperatures as the heating rate (*q*) increases. The determined activation energy values for the discontinuous precipitation of strengthening phases in the supersaturated 2017A alloy, determined by DSC using the Kissinger, Ozawa, and Boswell models, proved to be comparable. For example, the activation energy for the precipitation of the transitional phase θ″ was 89.94, 98.7, and 94.33 kJ/mol, respectively.

The lack of available literature data on the kinetics and activation energy of strengthening phase precipitation in the investigated 2017A alloy prevents a direct and unequivocal comparison of the obtained results with those of other authors. This limitation makes it difficult to assess the extent to which the observed properties align with trends reported in the literature, as potential differences in microstructure, processing conditions, and alloy composition may significantly impact the final material characteristics. However, similar dependencies have been observed in other studies on chemically similar alloys from the 2xxx series [10,12,13,16,36,38,40].

From an industrial perspective, the obtained results highlight the economic and environmental benefits of using recycled 2017A alloy instead of conventionally produced material. Secondary aluminum production requires only about 5% of the energy needed for primary aluminum extraction, leading to reduced raw material costs, lower CO_2_ emissions, and increased sustainability of the process. These factors confirm the industrial feasibility of using recycled aluminum in aviation, automotive, and structural applications.

In summary, this study provides new insights into the behavior of precipitates and the activation energy of strengthening phases in recycled 2017A alloy, demonstrating that the recycling process does not negatively affect the alloy’s microstructure or mechanical properties. The obtained results contribute to the further development of sustainable aluminum recycling technology and encourage further research on the long-term durability and environmental impact of recycled aluminum alloys. Future studies should focus on the influence of various secondary aluminum processing methods on microstructure and mechanical properties, as well as the optimization of heat treatment to further improve the operational parameters of 2xxx series alloys.

## 5. Conclusions

1. Microscopic observations (LM) and XRD studies showed that the microstructure of the recycled as-cast 2017A alloy consists of precipitates of intermetallic phases—θ-Al_2_Cu, β-Mg_2_Si, Al_7_Cu_2_Fe, Q-Al_4_Cu_2_Mg_8_Si_7_, and α-Al_15_(FeMn)_3_(SiCu)_2_—which crystalize mainly in the form of eutectics in the interdendritic regions of the solid α-Al solution. During solution heat treatment, the primary precipitates of the θ and Q phases were almost completely dissolved in α-Al. XRD and TEM studies of the aged specimens showed that the precipitation of the θ phase, and to a lesser degree, the Q phase, was responsible for the strengthening of the recycled 2017A alloy.
2. During natural and artificial aging of supersaturated 2017A alloys, an increase in Brinell hardness (HB) was observed with increasing aging time. The artificially aged alloy (175 °C) exhibited the highest hardness value (150.5HB). When aged at 120 °C, the hardness remained close to the value obtained during natural aging (approximately 128 HB).
3. The results of the conducted studies (DSC and XRD) show that the aluminum 2017A alloy obtained from recycled scrap exhibits a precipitation sequence consistent with that observed in the primary alloy: α-ss → GP/GPB zones → θ″→ θ′/Q′ → θ-Al_2_Cu/Q-Al_4_Cu_2_Mg_8_Si_7_. The maximum strengthening of the alloy results from the precipitation of metastable transition phases—θ″, θ′, and Q′. DSC analysis confirmed that despite the presence of trace elements such as Fe and Si, the kinetics of precipitation of metastable and stable strengthening phases did not undergo significant changes. Properly selected heat treatment parameters enabled the formation of a microstructure comparable to that of the primary alloy, indicating the potential for effectively utilizing recycled aluminum in applications requiring high mechanical properties.
4. The precipitation kinetics of the alloy was characterized by the DSC method under non-isothermal conditions. It was found that the heating rate q reduces the time to peak occurrence and affects the peak temperatures $T_{p}$ of precipitation of strengthening phases in the supersaturated 2017A alloy. The $T_{p}$ values of the exothermic/endothermic peaks in the DSC thermograms shift to higher temperatures as the heating rate q increases.
5. The activation energy values associated with the discontinuous precipitation of strengthening phases in the supersaturated 2017A alloy, determined by DSC methods using the Kissinger, Ozawa, and Boswell models, were found to be similar. For example, the activation energy for the precipitation of the θ″ transition phase was 89.94, 98.7, and 94.33 KJ mol^−1^, respectively.

## Figures and Tables

**Table 1:** Chemical composition of the 2017A aluminum alloy, weight%.

Alloy	Elements Content, wt%									
	Si	Fe	Cu	Mn	Mg	Cr	Zn	Zr	Ti	Al
**2017A**	0.49	0.22	4.01	0.56	0.72	0.063	0.20	0.17	0.077	balance

**Table 2:** The onset, offset, and peak temperature values of the main peaks observed in the DSC curve of the 2017A alloy, along with their corresponding reactions.

Peak	Reaction	Temperature, °C		
		Onset	Offset	$\mathrm{Top}\;\mathrm{of}\;\mathrm{Peak}\;(T_{p})$
A—exo	Formation of clusters	61.0	95.3	76.8
B—exo	GP1 zone formation	137.9	158.9	148.2
C—exo	GP2 zone formation	178.1	204.2	194.1
B′—exo	Dissolution of GP	-	214.8	-
D—exo	Formation of θ″	239.6	265.1	252.0
E—exo	Formation of θ′/Q′	273.2	311.1	290.6
C′—endo	Dissolution of θ″ and θ′	313.0	323.4	317.0
F—exo	Formation of θ	329.4	367.1	344.2
G—exo	Formation of Q	401.2	444.0	430.0
E′—endo	Dissolution of θ and Q	448	470.5	488
F′—endo	Dissolution of all phases	508.2	522.7	515.0
G′—endo	Dissolution of α-Al	619.0	659.3	648.6

**Table 3:** Variation in DSC top peak temperature (°C) with heating rate (°C min^−1^).

$\mathrm{Top}\;\mathrm{Peak}\;\mathrm{Temperature}\;T_{p}$, °C	Heating Rate q, °C min^−1^				
	5	10	15	20	25
**Vacancy cluster formation**	67.0	76.4	80.7	86.8	90.8
**GP1 zones**	135.0	150.4	157.4	166.8	169.6
**GP2 zones**	186.5	194.8	198.9	202.6	208.2
**Precipitation of θ″**	236.9	251.8	261.2	268.6	275.0
**Precipitation of θ′/Q′**	271.0	290.2	304.7	315.0	323.8
**Precipitation of θ**	330.0	344.2	353.8	362.3	364.7

**Table 4:** Values of activation energy precipitation of strengthening phase in the 2017A aluminum alloy determined by Kissinger, Ozawa, and Boswell methods.

Mathematical Models Used to Determine E_a_	Activation Energy Ea/kJ mol^−1^					
	Clusters	GPZ 1	GPZ 2	θ″	θ′/Q′	θ
**Kissinger**	64.3	61.3	133.6	89.94	72.5	132.2
**Ozawa**	70.1	68.4	141.4	98.7	81.9	142.5
**Boswell**	67.2	64.8	137.5	94.33	77.2	137.4

## Data Availability

The original contributions presented in this study are included in the article. Further inquiries can be directed to the corresponding author.
